# Leaf ionome signatures as drought tolerance indicators in chilli

**DOI:** 10.3389/fpls.2025.1673580

**Published:** 2025-11-14

**Authors:** Muddarsu Venkata Ramana, Subramanian Manivannan, Sujata Upadhyay, Palaniyandi Umadevi

**Affiliations:** 1 Department of Horticulture, Sikkim University, Gangtok, India; 2 Dr. Y.S.R. Horticultural University, Andhra Pradesh, Venkataramannagudem, India; 3 Department of Horticulture, Central University of Tamil Nadu, Thiruvarur, India; 4 Indian Council of Agricultural Research (ICAR) – Indian Agricultural Research Institute (IARI), Rice Breeding & Genetics Research Center, Aduthurai, Tamil Nadu, India

**Keywords:** chilli, drought, leaf ionome, PEG, hydroponics, mineral nutrition, climate change, water scarcity

## Abstract

Chilli (*Capsicum annuum* L.) cultivation is confined to warm and semi-arid regions where irrigation availability is often limiting production. Drought is one of the constraints of chilli cultivation. Hence, the development of drought-tolerant varieties is increasingly essential, and efforts are directed towards developing methods to understand chilli plant responses to water deficit. In this context, we used ionomics to study the drought tolerance of chilli. The ionome profiling of leaves using inductively coupled plasma mass spectrometry (ICP-MS) and integrating it with morpho-physiological and biochemical characteristics of eight cultivars using principal component and correlation analyses revealed the signature ionome mediating varying levels of drought tolerance. The accumulation of Ca, Cu, Mg, Fe, Mn, Mo, Ni, and Sn ions in leaves indicated them as markers for drought tolerance in chilli. This is a major step towards the identification of drought-tolerant cultivars using a simple and efficient technique in chilli. This method can serve as a simple, modern high-throughput tool in pre-breeding for selecting the drought-tolerant genotypes in crop improvement programmes.

## 1 Introduction

Chilli (*Capsicum annuum* L.) is one of the essential members of the family Solanaceae. Although India cultivates chilli on 0.915 million hectares, producing 1.872 million metric tons in 2016–2017, the low productivity of 2.25 t/ha, as compared to countries like Korea and Indonesia, whose productivity ranged from 4 to 6 t/ha (Paramaguru and Phanikumar, 2019), was a major problem. The primary reason is that chilli cultivation is confined to warm and semi-arid regions where irrigation availability is often limiting production (Dorji et al., 2005). Hence, the development of drought-tolerant varieties is increasingly essential. Drought, as a direct result of water shortage and increasing global temperatures due to climate change, is a serious threat to agricultural productivity and global food security. Traits that improve water use efficiency, the ability to withstand elevated temperatures, including heat tolerance and higher yield potential under stress, are desirable because these environmental challenges not only decrease the productivity of crops but also increase their susceptibility at a time when world population is expected to be 9.5 billion people by 2050, thereby increasing demands on food supplies (Valenzuela and Anderson, 2011). To combat these challenges, crop research has focused on enhancing the resistance of crops to abiotic stress and increasing resource use efficiency (Franco-Navarro et al., 2025). Mineral nutrients are essential to improve drought resistance through osmotic adjustment, energy metabolism, and antioxidative defence. Potassium and nitrate maintain stomatal regulation and osmolyte synthesis, whereas phosphate is needed for ATP generation (Khan et al., 2023). Chloride, a recently established beneficial macronutrient, enhances osmotic regulation and water use efficiency (Franco-Navarro et al., 2021, 2025). Drought tolerance involves the combination of avoidance through deep rooting, decreased transpiration and tolerance through osmotic adjustment, compatible solutes, and antioxidant activity (Ahanger et al., 2014; Khan et al., 2025). Many crops use both strategies, influenced by hormone and nutrient interactions with K^+^ and Cl^−^ supporting turgor, while root plasticity facilitates deep water extraction. Accordingly, mineral nutrition is one of the foundations of integrated drought resistance mechanisms, allowing for sustained plant growth in the presence of limiting water.

Efforts are directed towards a better understanding of chilli plant responses to water deficit. The basic approach to developing drought-tolerant genotypes is to select germplasm containing drought-adaptive traits. Our hydroponics study demonstrated the screening of eight chilli cultivars based on morpho-physiological characteristics and biochemical analysis (Muddarsu and Manivannan, 2017) using PEG-induced drought stress. Identifying and understanding the molecular mechanisms underlying drought stress is crucial for developing drought-tolerant varieties. Nowadays, many new-generation techniques are available to elucidate the molecular mechanism of a plant under particular stress. Comparing the ionome of different genotypes across multiple experiments will enhance the ability to identify drought stress and natural variants and allow the identification of classes of ionome profiles with common underlying physiological foundations (Baxter, 2010). Ionomics studies the entire mineral nutrients and trace elements found in an organism (Salt et al., 2008). Ionomics can capture information about the functional state under different developmental conditions and by biotic and abiotic factors. The ionome of industrially important crops can also serve as a geographical indication (Debbarma et al., 2021). Hence, we hypothesized that drought-tolerant cultivars maintain ionic homeostasis through the selective accumulation of key ions that enhance osmotic adjustment and antioxidative defence and that ionome signatures can serve as reliable indicators to distinguish tolerant from susceptible genotypes. This integrative approach provides a simple and high-throughput method for screening drought tolerance in chilli and other crops. Therefore, we used ionomics as a tool to tag the degree of drought tolerance by integrating the ionome profile with the morpho-physiological and biochemical characteristics exhibited by eight chilli varieties. Furthermore, we suggest the mechanism of drought tolerance in the selected tolerant and susceptible varieties of chilli using the ionome dynamics along with marker ions. The method discussed in this paper can serve as a simple and high-throughput technique to identify drought tolerance in any crop.

## 2 Materials and methods

### 2.1 Drought stress induction

Seeds of eight chilli (*C. annuum* L.) varieties were obtained from institutions in conformity with the Protection of Plant Varieties and Farmers’ Rights Act, 2001 (PPVFR Act, 2001), Government of India (GoI). Arka Lohit and Arka Mohini were procured from the Indian Council of Agricultural Research–Indian Institute of Horticultural Research, Bengaluru (ICAR-IIHR); LCA-353, LCA-334, LCA-625, G4, and CA-960 were from Horticulture Research Station (HRS), Dr. YSR Horticultural University, Lam, Guntur; Dalle Khurasani was procured from Sikkim University. Drought stress was applied in a hydroponic system based on Muddarsu and Manivannan (2017) at the Growth and Development Laboratory, Sikkim University. Seeds were surface sterilized with 70% ethanol for 1 minute, followed by HgCl_2_ for 3 minutes, and rinsed three times with distilled water. Seedlings were raised in portrays containing a cocopeat–perlite mixture (1:1 v/v), with one seed sown per cell. The trays were maintained in a controlled growth laboratory at 28°C under regular irrigation, and seedlings were grown for 14 days. The 14-day-old seedlings were then grown in trays filled with Hoagland’s solution (Hoagland and Arnon, 1950) under controlled environment conditions at 16-h photoperiod, 70 μmol m^−2^ s^−1^ of photosynthetic photon flux density (PPFD), 28°C, 60%–70% Relative humidity (RH), and medium pH maintained at 5.5–6.0 in a culture room enabled with photosynthetically active radiation (PAR) lights and temperature control. Three days after the transplant of seedlings, different drought stress levels were formed using PEG 6000 (0%, 5%, 10%, 15%, and 20%). The medium was prepared fresh and replaced every seventh day. The osmotic potential (Ψs) for the PEG 6000 solutions was calculated according to Michel and Kaufmann (1973). At 28°C, the osmotic potential of PEG 6000 solutions was approximately −0.38, −0.82, −1.33, and −1.91 MPa for the concentrations 5%, 10%, 15%, and 20%, respectively, calculated as follows:


$$\Psi s(MPa)=-0.1\times [(1.18\times 10^{-2})C+(1.18\times 10^{-4})C^{2}-(2.67\times 10^{-4})C\cdot T-(8.39\times 10^{-7})C^{2}\cdot T]$$

where Ψs is the osmotic potential (MPa), C is the PEG concentration (g L^−1^ ≈ g kg^−1^ water), and T is the absolute temperature (K).

On the 30th day, leaf samples were harvested from non-stress plants (0% PEG) and plants under stress conditions (5%, 10%, and 15% PEG, representing 50, 100, and 150g L^−1^, respectively) for ionome determination.

### 2.2 Sample preparation and ionome profiling

The leaves were washed with tap water to remove the adhered dust particles, followed by 1% Teepol solution for 3 minutes. They were then washed with 0.1N HCl for 3 minutes and washed three times with double-distilled water; the samples were dried in a hot air oven at 60°C for 24 hours. The dried samples were processed into a powder using a Wiley mill (Model 4; Thomas Scientific, Swedesboro, New Jersey, USA) and stored in an airtight container with a label.

The microwave digestion of the samples was performed using the multiwave digestion system (Multiwave 3000; Anton Paar, Ashland, Virginia, USA) as per the following conditions: Infrared (IR), 190°C; power, 1,200 W; ramp, 5 minutes; rate, 0.3bar sec^−1^; hold, 8 minutes; sample size, 0.5g; acids used, 5 mL HNO_3_ and 1 mL HCl. Digested samples were then cooled, and the volume was made up to 50 mL with double-distilled water. Analysis of the samples was carried out using inductively coupled plasma mass spectrometry (ICP-MS; NexION 300X; PerkinElmer, USA) system (Chettri et al., 2018) fitted with a GemTip Cross-Flow II nebulizer (model N0770546; PerkinElmer, USA) at Plant Nutrition and Ionome Laboratory, Department of Horticulture, Sikkim University, Sikkim, India. Twenty-eight elements were profiled: silver (Ag), aluminium (Al), boron (B), barium (Ba), calcium (Ca), chromium (Cr), caesium (Cs), copper (Cu), iron (Fe), gallium (Ga), Iodine (I), potassium (K), lithium (Li), magnesium (Mg), manganese (Mn), molybdenum (Mo), sodium (Na), nickel (Ni), phosphorus (P), lead (Pb), rubidium (Rb), sulfur (S), silicon (Si), titanium (Ti), vanadium (V), zinc (Zn), zirconium (Zr), and mercury (Hg). Ten morpho-physiological parameter—shoot length, root length, number of leaves, number of internodes, leaf area/plant, shoot fresh weight, root fresh weight, shoot dry weight, root dry weight, and root to shoot dry weight—and one biochemical parameter (proline content) were used to correlate the ionome using principal component analysis (PCA) to attribute it to the level of drought tolerance. The method for estimating the morpho-physiological and biochemical parameters is detailed in Muddarsu and Manivannan (2017).

### 2.3 Statistics

A completely randomized block design (CRD) was used. Under each factor, i.e., control, stress, and genotypes, three replications were maintained. From each replication, three biological replicates were taken for ionome analysis. The PCA was performed after auto-scaling the data of 39 parameters of eight chilli cultivars. The parameters were 28 elements, 10 morpho-physiological parameters, and one biochemical parameter.

The principal components were extracted according to the Kaiser (Kaiser, 1960) criterion (eigenvalues > 1), and the cumulative variance explained by the initial components was taken as significant in the variability description of the dataset. PCA explained more than 60% of the variance, with due contribution of different ions to drought tolerance through positive morpho-physiological traits established using the correlation analysis, which unlocked the key for the identification of marker ions.

## 3 Results

### 3.1 Ionome pattern under control (non-drought) condition

**Figure 1:**
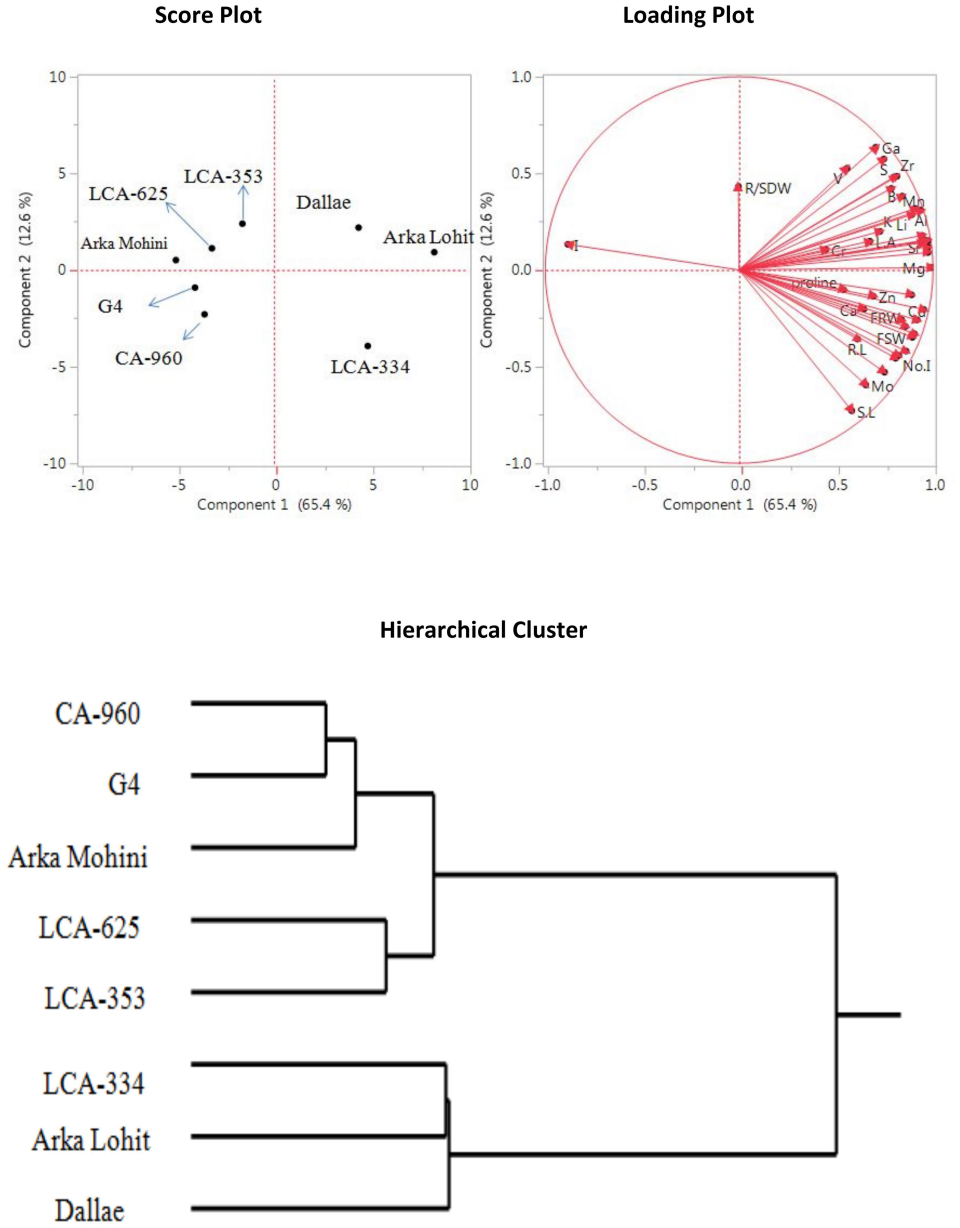
Principal component analysis (PCA) and hierarchical clustering of chilli cultivars under control condition showing genotypic variation and trait association.

Under the control condition (0% PEG), principal component analysis data (**Figure 1**) revealed that the first two principal components had eigenvalues greater than 1, accounting for 78.0% of the total variation. It was found that the first principal component contributed 65.4% whereas the second principal component contributed 12.6% of the total variation analysed. The physiological traits root dry weight, shoot fresh weight, shoot dry weight, fresh root weight, number (no.) of internodes, no. of leaves, leaf area, root length, shoot length, proline, and root to shoot dry weight and the concentrations of elements Co, Cu, P, Sn, Ni, Zn, Ca, and Mo had positive contribution to PC1. Ga, S, V, Cs, Zr, Ba, B, Mn, Al, Li, K, Ce, Fe, Ti, Na, I, Sr, Cr, Rb, Mg, leaf area, and root to shoot dry weight positively contributed to PC2. Mg, Ti, Sr, Rb, Fe, Na, Ce, Al, Mn, Li, B, Zr, Cs, Ba, S, K, Ga, V, Cr, leaf area, and root to shoot dry weight contributed to both PC1 and PC2. Score plot and hierarchical cluster analysis revealed that eight capsicum cultivars could be separated into three groups. Arka Lohit, LCA-334 and Dalle Khurasani cultivars were combined into one group. The second group of cultivars comprised LCA-353 and LCA-625, and the third group had CA-960, G4, and Arka Mohini.

### 3.2 Ionome pattern under drought conditions

#### 3.2.1 5% PEG 6000 drought condition

**Figure 2:**
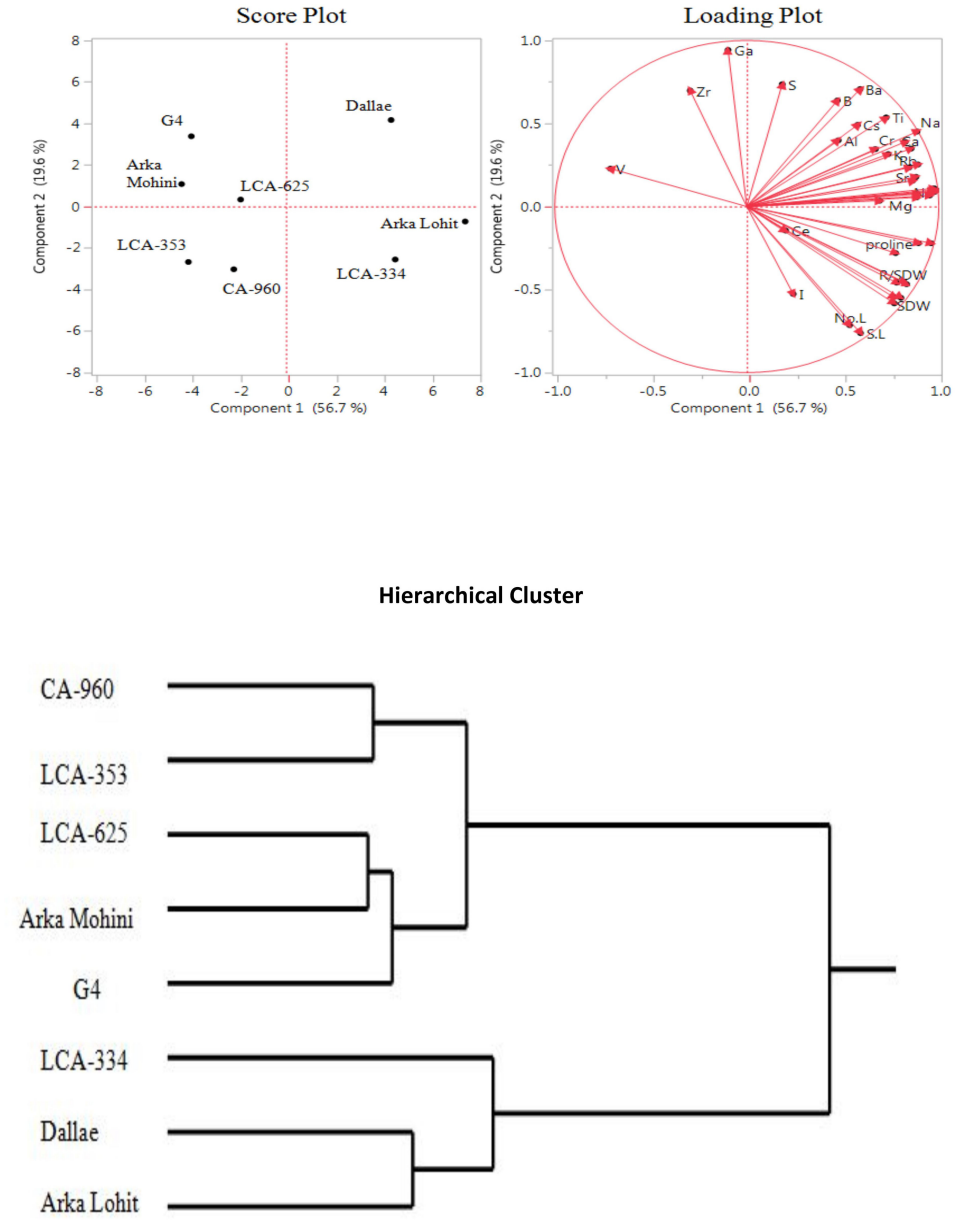
Principal component analysis (PCA) and hierarchical clustering of chilli cultivars under the 5% PEG-induced drought condition showing genotypic variation and trait association.

Under the 5% PEG-induced drought stress condition, principal component analysis data (**Figure 2**, **Table 1**) revealed that the first two principal components had eigenvalues greater than 1, accounting for 76.3% of the total variation. It was found that PC1 contributed 56.7% whereas PC2 contributed 19.6% of the total variation. Cu, Ni, Fe, Co, root fresh weight, P, proline, Mn, Na, Mg, Sn, Sr, Mo, Ca, Rb, root dry weight, Li, root to shoot dry weight, shoot fresh weight, no. of internodes, leaf area, root length, shoot dry weight, K, Ti, Zn, Cr, shoot length, Ba, Cs, no. of leaves, Al, B, I, Ce, and S contributed positively in descending order to PC1; V, Zr, and Ga elements contributed negatively to PC1. However, Ga, S, Ba, Zr, B, Ti, Cs, Na, Al, Li, Ca, Cr, K, Mn, Rb, V, Sr, Mo, Fe, Cu, Co, Ni, Sn, P, Mg, and Zn positively contributed to PC2; all physiological characteristics and elements I and Ce negatively contributed to PC2. Score plot and hierarchical cluster analysis revealed that eight capsicum cultivars could be separated into three groups. Dalle Khurasani, Arka Lohit, and LCA-334 could be grouped together and positively contributed to PC1. The second group of cultivars consisting of LCA-625, Arka Mohini, and G4 positively contributed to PC2. Cultivars CA-960 and LCA-353 formed the third group in the quadrant where PC1 and PC2 were negative.

**Table 1:** Correlation of growth parameters and ionome signatures across various levels of drought stress.

PEG level (in %)	0		5		10		15		20	
Growth parameters	Positive	Negative	Positive	Negative	Positive	Negative	Positive	Negative	Positive	Negative
Shoot length	P*, V, Li, Ti	I	Cs**, Cr, Ba, Na, Zn, Mn	V	Ba**, Ca**, P, Mg, Ga, Fe, Mn, Cu, K, Rb,	I, S, V	Co**, Ni**, Cu, Sn, Al, Rb, Mg, Cr, Ce, Ga, Mo, Mn, Fe, Ba, Sr, Zr, K,	S, I	Ba**, Ga**, Cu**, Fe**, Mn, Zr, Mg, Ca	Cr, Ni, V, Zn, S, Sr, Sn, Ti, I, Co
Root length	Zr, Mn, Cs, Ba, B, Al, S, Ga, V, Ti, Fe		Cr**, P**, Mg**, Fe, Mn, Na, Zn, Cs, Cu, Sn, Al, NI, Ba, Co. Ti		Ba**, Ca**, P**, Mg, Ga, Fe, Mn, Cu, B, K, Rb	V, S	Al**, Cu**, Ce**, Mo**, Co**, Ni**, Fe**, Mg**, K**, Sn**, Rb**, Cr, Ba, Sr, Mn, Cs, Na, Zr, Ca,	S, I	Ba**, Ga**, Zr**, Cu**, Fe**, Cs**, Mn**	Cr, Zn, V, Ni, S, Sr, Sn
Number of leaves	Li**, Ti**, V**, P, Sr, Al, Na, Fe, Mg, Zr, S, Mn,	I	Al**, Cr**, Ba**, Na, Mg		P, Ba, Ca	I, V, Ni, Li	Co, Ni, Cu, Al, Rb, Mg, Sn, Ce	I, S, Zn		V, I, Ti, K
Shoot dry weight	P**, Ti**, Fe**, Mg**, Sr**, Li**, Co**, Cu, Rb, Ce, Al, Mn, Sn, K	I	Cr**, Zn**, Mg**, P**, Fe**, Al**, Mn, Cu, Na, Ba, Co, Ni, Cs, Ca, Sn		B**, Ba, P, Ca, Ga,	I	Mo**, Ce**, Ba**, Sn**, Al**, Zr**, Co, Na, Ni, Cu, Fe, Ga, Rb, K, Cr, Mg, Cs, Sr	S, I	Ba, Ga	Zn, S, Sr, V, Ni, Rb, Ti
Root dry weight	Ti**, SR**, Li**, P**, Mg**, Na**, Fe**, Ce**, Al**, Rb**, Co**, Mn, Cu, Zr, B, V, K Sn, Ba,	I	Cr,** Mg**, P**, Fe**, Na, Mn**, Ca**, Cu**, NI, Co, Al, Zn, Ti, Ba, Sn, K, Cs, Sr, Li,		Ba**, Ca**, P**, Mg**, Fe, Cu, Ga, Mn, K, B, Rb, Sn, Mo, Na	I, S, V	Al**, Mo**, Ce**, Cu**, Co**, Sn**, Ni**, Fe**, Ba, Rb, Mg, Zr, K, Cr, Na, Sr, Mn, Cs, Ga	S, I	Ba**, Ga**, Cu**, Fe**, Mn, Zr, Mg, Ca	Cr, Zn, S, Sr, V, Ni, Ti, I
Proline	Ca, Ba		Sn**, Cu**, Na**, Mn**, Ni**, Cs**, Co** Mo**, Rb**, Fe**, Sr**, Li**, P, Ca, Ti, Zn, Ba, Mg, K	V, Zr	B**, Ba, P, Ca, Mn, Mg, Ga, Cu	I, V, S, Ni	Rb, Co, Zr, Ga, Ni, Mg, Cr, Ba, Cu, Al, Sr, Ca, Mn, P	I, S	Zr, Cs, Ba, Ga, Na	Cr, Ni, V, S, Sn, Sr, Zn.

(*) means the correlation is significant at the 0.05 level (p < 0.05), (**) indicate significance at the 0.01 level (p < 0.01).

#### 3.2.2 10% PEG drought condition

**Figure 3:**
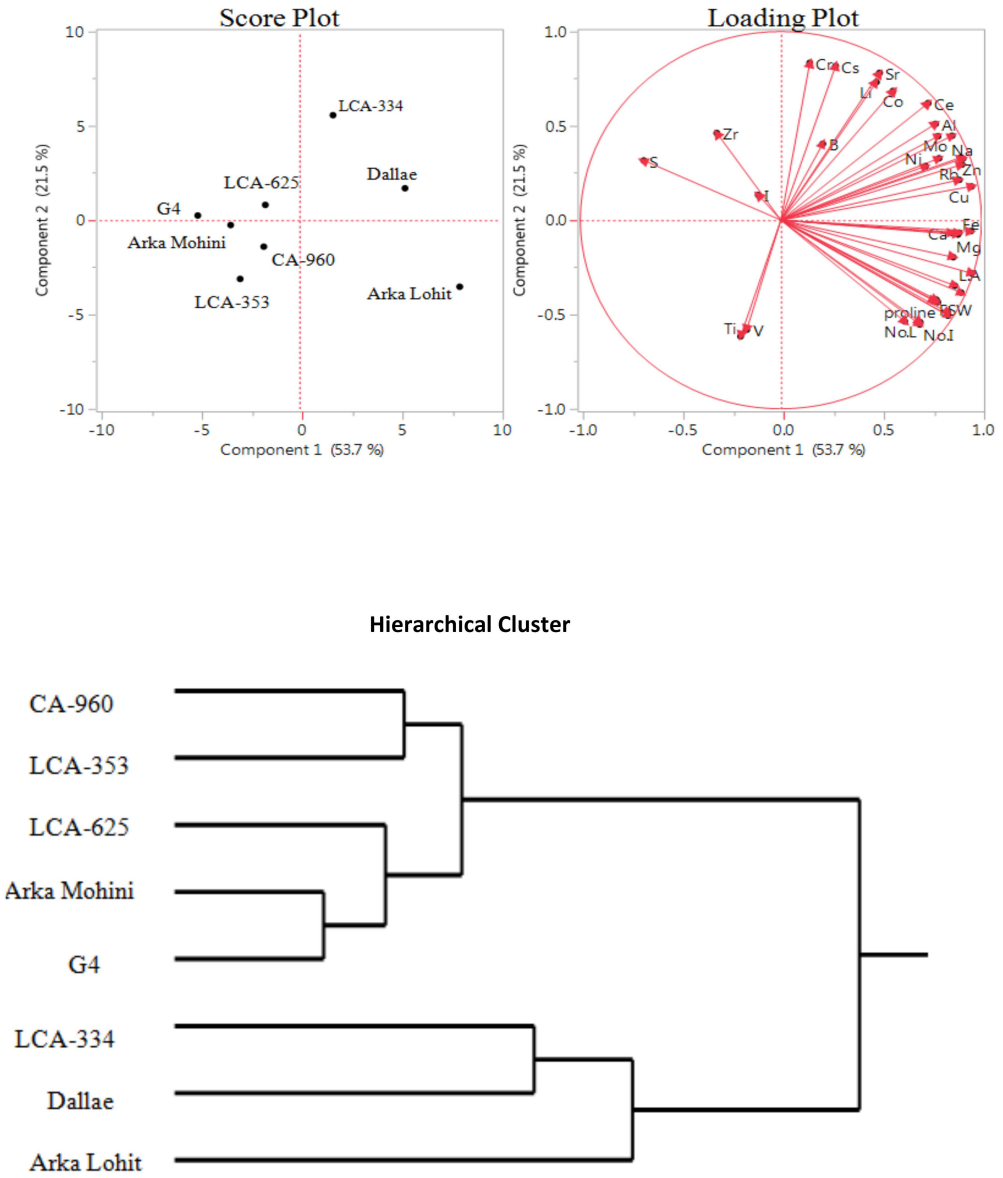
Principal component analysis (PCA) and hierarchical clustering of chilli cultivars under the 10% PEG-induced drought condition showing genotypic variation and trait association.

Under the 10% PEG-induced drought stress condition, principal component analysis data (**Figure 3**; **Table 1**) revealed that the first two principal components had eigenvalues greater than 1, accounting for 75.2% of the total variation. It was found that PC1 contributed 53.7% whereas PC2 contributed 21.5% of the total variation. Root to shoot dry weight, Cu, Fe, K, Rb, root dry weight, Na, Zn, Ba, Mn, leaf area, Mg, Ca, Mo, root length, shoot length, root fresh weight, P, Sn, proline, shoot fresh weight, Al, shoot dry weight, Ce, Ni, no. of leaves, no. of internodes, Ga, Co, Sr, Li, Cs, B, and Cr positively contributed in descending order to PC1; S, Zr, Ti, V, and I negatively contributed to PC1. Cr, Cs, Sr, Li, Co, Ce, Al, Zr, Mo, Sn, B, Na, P, K, S, Rb, Ni, Zn, Cu, and I positively contributed in descending order to PC2; Ti, V, no. of internodes, no. of leaves, Ga, root length, shoot length, root fresh weight, proline, shoot dry weight, shoot fresh weight, root dry weight, leaf area, root to shoot dry weight, Mg, Mn, Ca, Ba, Fe, and I negatively contributed to PC2. Score plot and hierarchical cluster analysis revealed that eight capsicum cultivars could be separated into three groups. Dalle Khurasani, LCA-334, and Arka Lohit could be grouped together and positively contributed to PC1; the second group of cultivars, LCA-625, Arka Mohini, and G4, fell near to origin. Cultivars CA-960 and LCA-353 formed the third group in the quadrant where PC1 and PC2 were on the negative side.

#### 3.2.3 15% PEG drought condition

**Figure 4:**
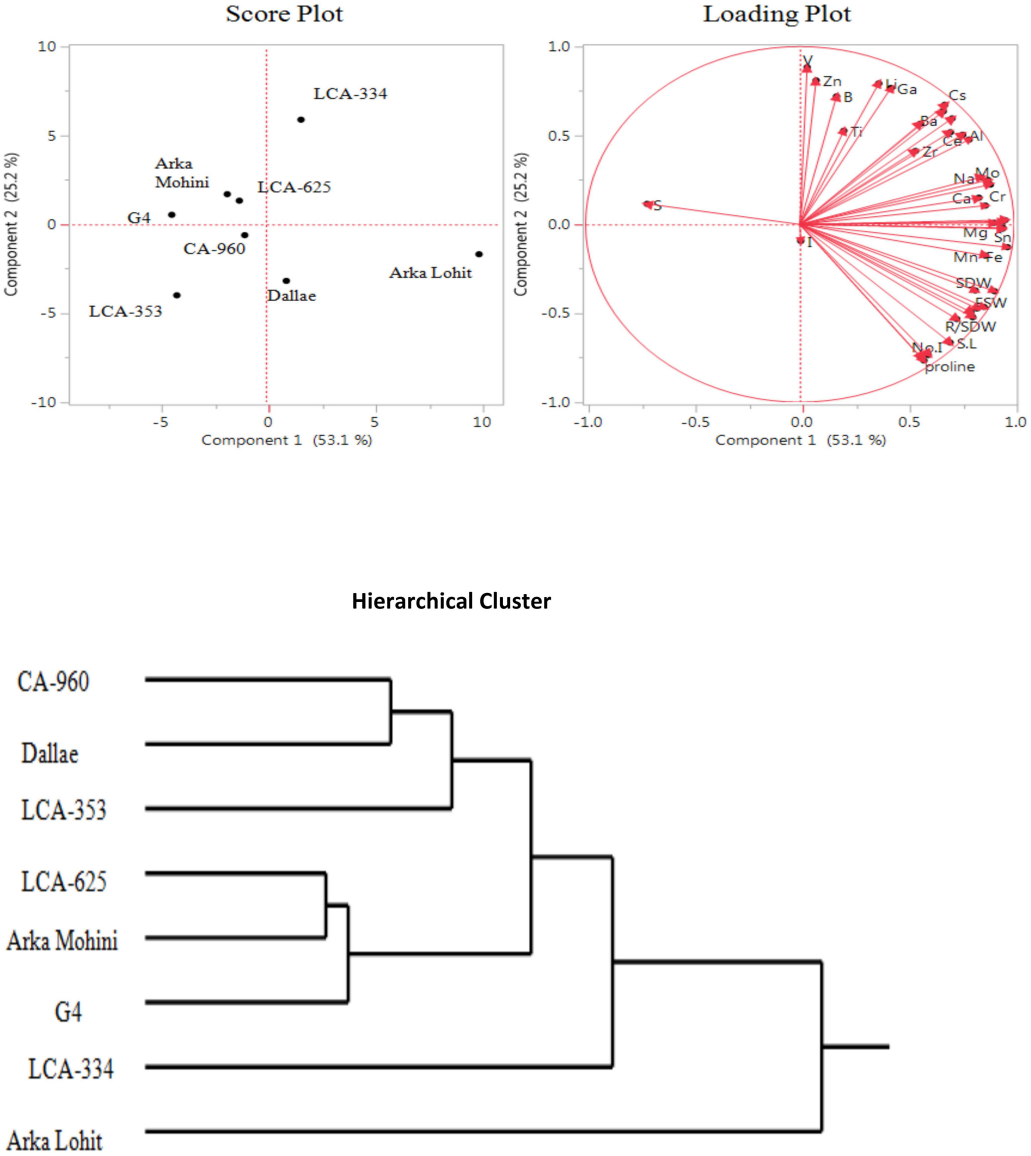
Principal component analysis (PCA) and hierarchical clustering of chilli cultivars under the 15% PEG-induced drought condition showing genotypic variation and trait association.

Under the 15% PEG-induced drought stress condition, principal component analysis data (**Figure 4**; **Table 1**) revealed that the first two principal components had eigenvalues greater than 1, accounting for 78.3% of the total variation. It was found that PC1 contributed 53.1% whereas PC2 contributed 25.2% of the total variation. Fe, Cu, Sn, Co, Ni, root fresh weight, Mg, K, Mo, Cr, Mn, root length, Na, Ca, root dry weight, shoot dry weight, root to shoot dry weight, shoot fresh weight, Ce, Al, leaf area, Rb, Sr, shoot length, Cs, Ba, no. of internodes, no. of leaves, proline, P, Zr, Ga, Li, Ti, B, Zn, V, and I contributed positively in descending order to PC1. V, Zn, Li, Ga, B, Cs, Ba, Rb, P, Ti, Sr, Al, Ce, Zr, Na, Mo, K, Ca, S, Cr, Cu, Co, and Mg positively contributed to PC2 in descending order; no. of leaves, proline, no. of internodes, shoot length, leaf area, root to shoot dry weight, shoot fresh weight, root dry weight, root length, root fresh weight, shoot dry weight, Mn, Fe, I, Ni, and Sn contributed negatively to PC2. Score plot and hierarchical cluster analysis revealed that eight capsicum cultivars could be separated into three groups. Arka Lohit and LCA-334 cultivars could be grouped together and positively contributed to PC1. LCA-625, G4, and Arka Mohini fell near to origin and formed the second group of cultivars. Cultivars CA-960, LCA-353, and Dalle Khurasani formed the third group and were placed where the below-average values of PC1 and PC2 were plotted.

#### 3.2.4 20% PEG drought condition

**Figure 5:**
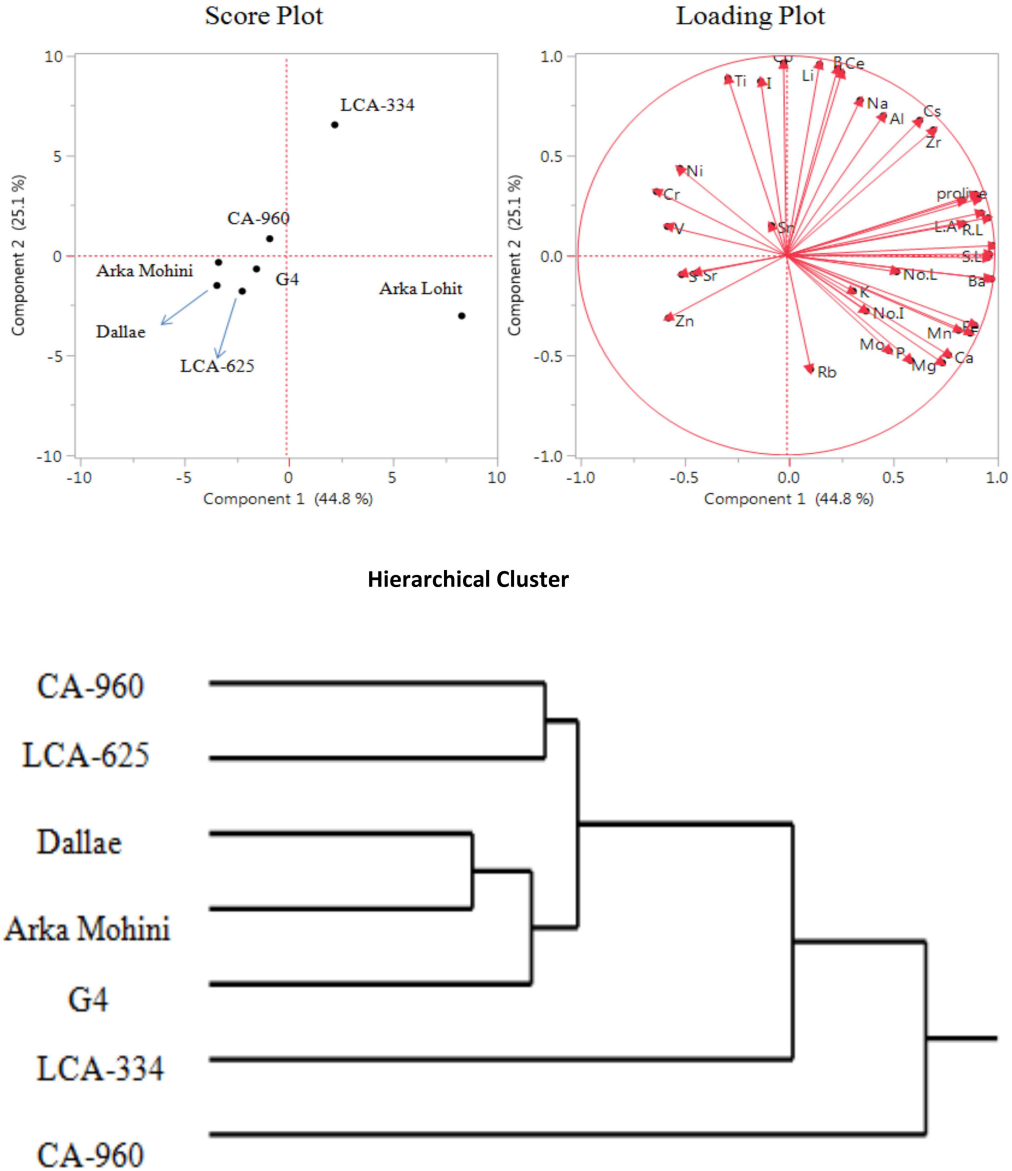
Principal component analysis (PCA) and hierarchical clustering of chilli cultivars under the 20% PEG-induced drought condition showing genotypic variation and trait association.

Under the 20% PEG-induced drought stress condition, the LCA-353 cultivar did not survive. Hence, LCA-353 was designated as highly susceptible to drought stress. Principal component analysis (**Figure 5**; **Table 1**) revealed that the first two principal components had eigenvalues greater than 1, accounting for 69.9% of the total variation. It was found that PC1 contributed 44.8% whereas PC2 contributed 25.1% of the total variation. Root dry weight, Ba, shoot length, Ga, root length, root to shoot dry weight, root fresh weight, shoot dry weight, shoot fresh weight, Cu, Fe, leaf area, proline, Mn, Ca, Mg, Zr, Cs, P, no. of leaves, Mo, Al, no. of internodes, Na, K, Ce, B, and Li positively contributed in descending order to PC1; Cr, V, Zn, Ni, S, Sr, Ti, I, Sn, Co, and Rb negatively contributed to PC1. Co, Li, B, Ce, Ti, I, Na, Al, Cs, Zr, Ni, Cr, shoot fresh weight, shoot dry weight, proline, root fresh weight, root length, leaf area, Sn, V, root dry weight, and shoot length positively contributed to PC2; Rb, Mg, P, Ca, Mo, Fe, Mn, Cu, Zn, no. of internodes, K, Ba, Ga, S, Sr, no. of leaves, and root to shoot dry weight negatively contributed to PC2. Score plot and hierarchical cluster analysis revealed that seven capsicum cultivars could be separated into two groups, viz. Arka Lohit is on the extreme positive side of PC1, followed by LCA-334 as a one group. All other cultivars fell near to origin and formed another group.

## 4 Discussion

The present study analysed eight chilli cultivars for their ionome and physiological trait patterns under normal and chronically imposed osmotic stress (control up to 20% PEG 6000). This study demonstrated the ability of PCA to deconstruct the complex interplay among ionomic composition, morpho-physiological traits, and drought tolerance in chilli cultivars. We only extracted components with eigenvalues greater than 1 (Kaiser criterion) in order to ensure that only statistically meaningful dimensions were retained, hence maximizing the part of variation contained in the dataset explained (Jolliffe, 2002). Across control and the three differing degrees of PEG that induced drought stress, the first two principal components continuously indicated a large fraction of the overall variance (20% PEG, 69.9%; 15% PEG, 78.3%). Such large fractions of explained variance suggest that the chosen ionomic and physiological traits represent sufficient information to accurately reflect the underlying variability of stress response.

### 4.1 Ionome pattern under control (non-drought) condition

Under non-stress conditions, 65.4% of the variance was explained by PC1 [dominated by biomass parameters (shoot and root weight, leaf area, and no. of internodes), along with proline and essential macronutrients Ca, P, Mo, Zn, Cu, and Ni]. These characteristics reflect the inherent physiological “vigour” of these genotypes and emphasize that cultivars can be clearly distinguished in terms of growth and nutrient uptake potential under conditions where water supply is not limiting. A similar conclusion was reported in other crops, where biomass and ion homeostasis rank as the first contributors for clustering of genotypes by PCA under non-stress conditions (Broadley et al., 2007; Huang and Salt, 2016).

### 4.2 Ionome pattern under drought conditions

In the 5% PEG, PCA revealed Dalle Khurasani, Arka Lohit, and LCA-334 as drought-tolerant and CA-960 and LCA-353 as susceptible. Essential elements Cu, Fe, Mn, P, Ca, Mg, and K and non-essential elements Na, Ni, Co, Li, Sr, Rb, Sn, and Ti contributed and correlated positively (**Figure 2**). Hence, they may be considered elements that contribute to drought tolerance under the 5% PEG-induced drought stress condition. In the 10% PEG, Arka Lohit, Dalle Khurasani, and LCA-334 were considered drought-tolerant cultivars, and CA-960 and LCA-353 were considered susceptible cultivars. Since essential elements K, Cu, Zn, P, Fe, Mn, Mg, and Mo and non-essential elements Rb, Na, Sn, Ba, Al, and Ce contributed to PC1 (**Figure 3**), they were considered elements that contribute to drought tolerance under the 10% PEG-induced drought stress condition.

In the 15% PEG, Arka Lohit was considered a drought-tolerant cultivar, followed by LCA-334, whereas CA-960, LCA-353, and Dalle Khurasani were considered susceptible cultivars. Since essential elements K, Cu, Mg, Ca, Fe, P, Mn, and Mo and non-essential elements Rb, Co, Ce, Al, Ni, Cs, Sn, Na, Sr, Cr, and Ba contributed to PC1 (**Figure 4**), they were considered elements that contribute to drought tolerance under the 15% PEG-induced drought stress condition. Under the 20% PEG-induced drought stress condition, LCA-353 could not survive and was designated as highly susceptible to drought stress. Arka Lohit was found to be tolerant, followed by LCA-334 (**Figure 5**). The correlation analysis with morpho-physiological characteristics and the ions across the germplasm and treatments proved similar results to those of the PC analysis (**Table 1**).

### 4.3 Ionome indicators for drought tolerance

Based on PCA, Arka Lohit fell under the quarter where all major essential elements Mg, Fe, Cu, Mn, Mo, Ca, P, and K and some of the non-essential elements Rb, Co, Ce, Al, Ni, Cs, Sn, Na, Sr, Cr, and Ba showed positive correlation under all drought stress conditions. In addition, LCA-334 also fell under the quarter where maximum elements had a positive association. Hence, Arka Lohit was found to be highly tolerant, followed by LCA-334. Under the 20% PEG stress condition, LCA-353 could not survive, and it was found to be susceptible. Considering all the stress conditions, the essential elements Fe, Mg, Mn, Mo, P, K, Ca, and Cu and non-essential elements Rb, Al, Na, Ni, Ba, and Sn were responsible for the drought tolerance of Arka Lohit and LCA-334.

### 4.4 Ionome dynamics upon drought between the tolerant and susceptible cultivars

**Figure 6:**
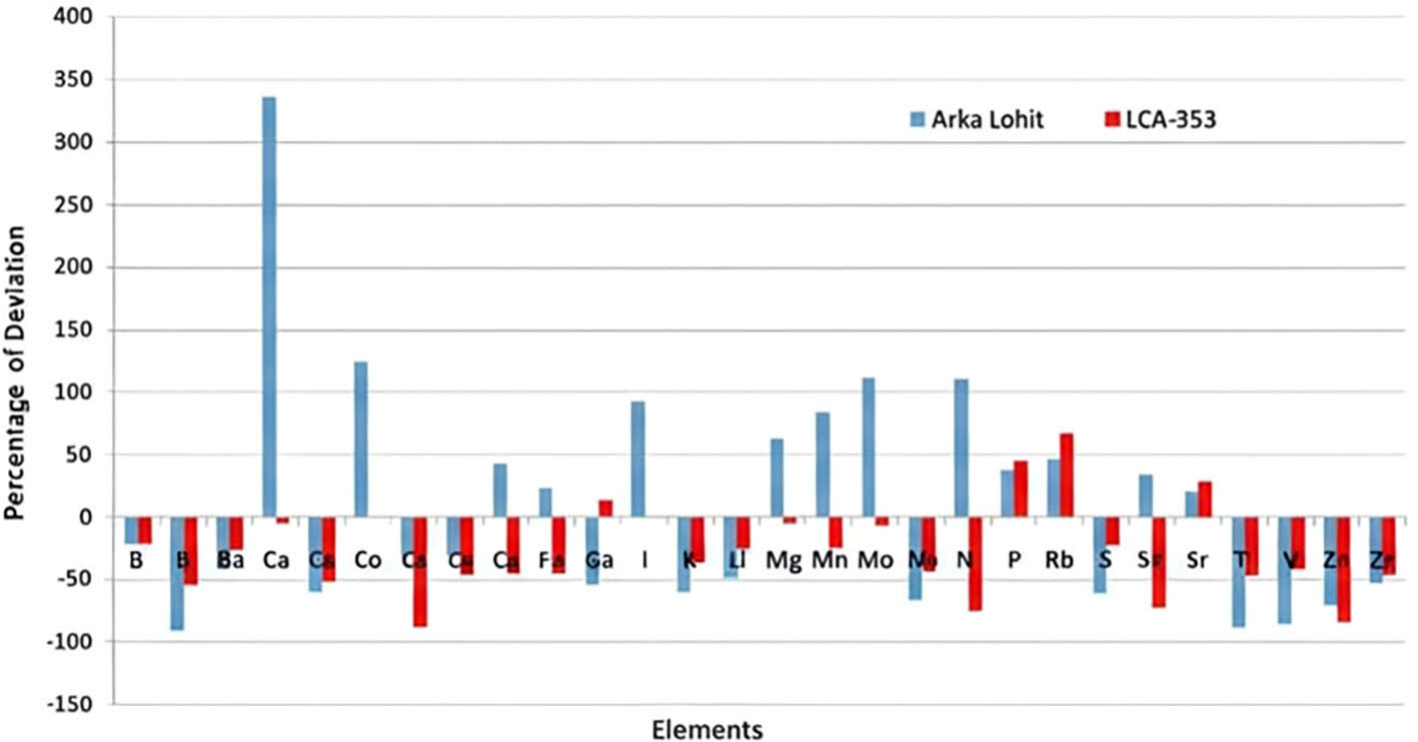
Comparative percentage of deviation of element concentration between drought-tolerant (Arka Lohit) and susceptible (LCA-353) chilli cultivars.

**Figure 7:**
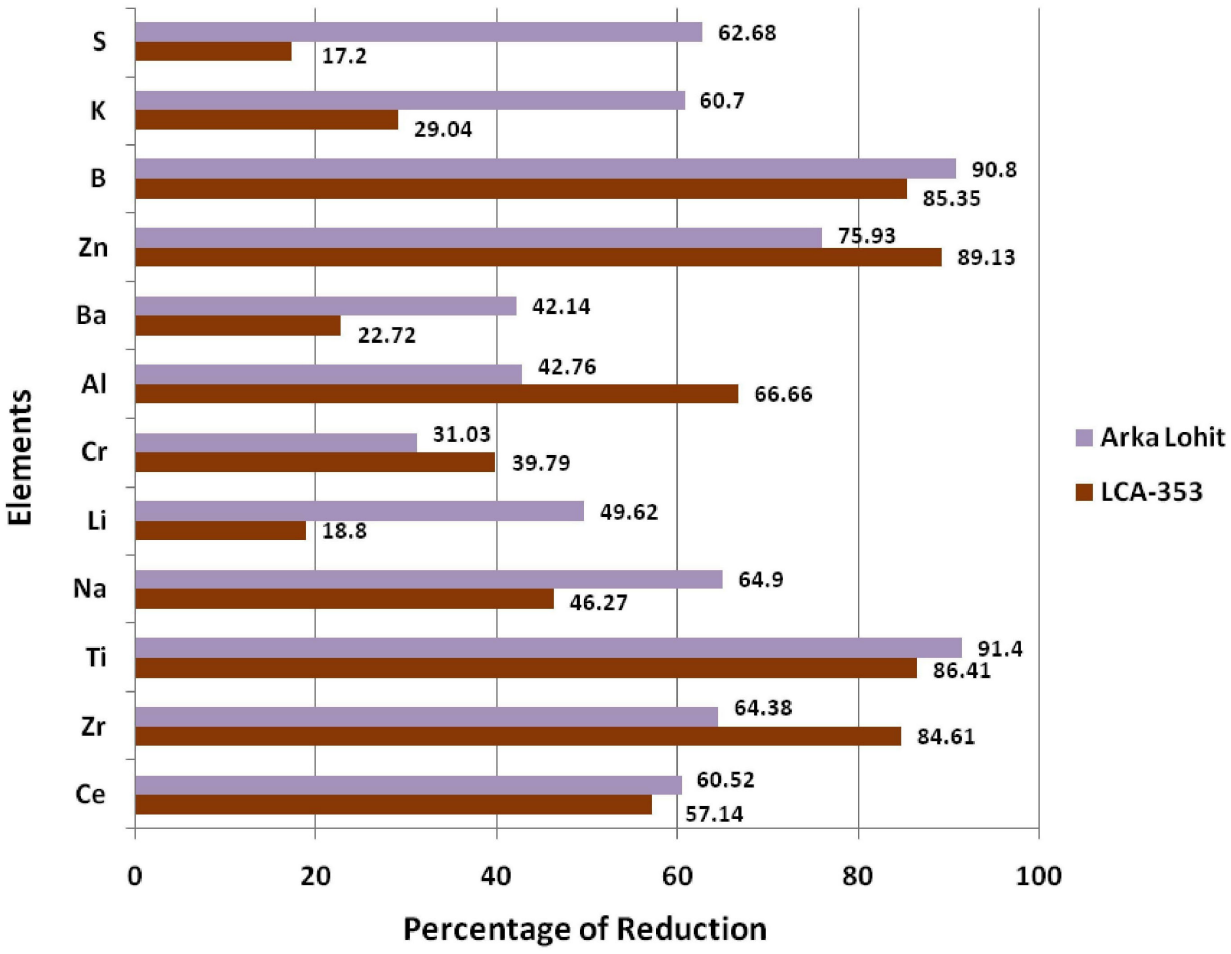
Comparative ionome reduction status between tolerant (Arka Lohit) and susceptible (LCA-353) cultivars under drought stress.

To further understand the mechanism of tolerance, the ionome dynamics were studied between the tolerant (Arka Lohit) and susceptible (LCA-353) cultivars under the 15% PEG-induced drought condition, which was the maximum tolerable limit of stress for the susceptible, as it did not survive under the 20% PEG condition. The concentrations of P, Rb, and Sr increased (**Figure 6**) under stress conditions in both drought-tolerant and drought-susceptible cultivars compared to the control, suggesting that these elements are drought-responsive in chilli. The percentage of ionome reduction under stress conditions compared to control in both Arka Lohit and LCA-353 is shown in **Figure 7**. The comparative ionome dynamics showed that the concentrations of Ca, Cu, Fe, Mg, Mn, Mo, Ni, and Sn increased under stress condition as compared to control in drought-tolerant cultivar (Arka Lohit), whereas in the drought-susceptible cultivar (LCA-353), these elements were reduced (**Figure 6**), suggesting that they were the primary elements responsible for drought tolerance.

In plant stress signalling, calcium serves as a known secondary messenger. Rapid changes in cytosolic Ca^2+^ stimulate downstream signalling cascades and the induction of drought-responsive genes. Recent research has indicated that an exogenous application of Ca (e.g., Ca(NO_3_)_2_) could ameliorate cell injury and alleviate the response to drought by promoting ion balance and the expression of stress-associated genes (Malyukova et al., 2022). Likewise, prohexadione-calcium treatment in wheat was reported to entail water balance and antioxidant metabolism improvement and subsequent enhancement of drought tolerance (Zhang, 2024).

In addition, copper contributes to the improvement of structural defence systems against drought. Recent data have indicated that foliar applications of Cu (CuSO_4_, Cu nanoparticles, or CuS) minimized soybean drought-induced biomass and reproductive reductions by promoting flowering and pod set development (Zhang, 2024). Moreover, the Cu deficiency was associated with a decreased level of lignin deposition and an alteration of the xylem structure in poplar, indicating its requirement for both lignification and cell wall metabolism (Cameron, H et al., 2023). Copper plays a significant role in lignification and cell wall metabolism, which can be enhanced by drought, and it increases structural defences. Higher Ca and Cu contents in the tolerant cultivar could therefore contribute to increased mechanical strength and stress signalling capability.

Fe is vital for photosynthesis and reactive oxygen species (ROS) detoxification. Recent findings have supported the idea that Fe nanoparticles (FeNPs) may mitigate drought stress by promoting antioxidant enzyme activity and chlorophyll retention under water deficit (Yılmaz, 2024). The higher Fe accumulation in Arka Lohit thus explained its better antioxidant ability over LCA-353.

Mg is essential to chlorophyll, the activation of enzymes, and metabolic balance. In tomato, magnesium-polyphenolic bio-stimulants have been recently demonstrated to exert a drought stress-promoting mechanism involving improved photosynthetic efficiency and osmoregulation (Hamedeh et al., 2022). High Mg in the tolerant cultivar probably promoted photosynthetic efficiency and carbon partitioning under stress, which would enhance drought response.

Mn plays a role in photosystem II, as a cofactor for enzymes and oxidative stress scavengers. Under drought, the study of mineral nutrients showed that plants absorb little Mn because the roots’ absorbing ability decreases in many crops, unless the plant actively maintains it (Lilay et al., 2024). Mn enrichment in Arka Lohit signifies that it has a function in maintaining photosynthetic integrity and oxidative homeostasis upon water stress.

The presence of higher contents of Mg, Mn, and Fe in the tolerant cultivar upon drought stress suggested improved photosynthetic mechanisms than in the susceptible cultivar to cope with the induced drought.

Mo is referred to as having an important role in nitrogen metabolism and antioxidative pathways. Micronutrient reviews have highlighted the role of Mo and the requirement for nitrate reductase and sulfur metabolism under stress conditions (Khan et al., 2023). The addition of molybdenum mitigated the negative effects of drought stress and enhanced water use efficiency, contributed by involving the enzyme system of N metabolism, S metabolism, and protein synthesis (Waraich et al., 2011).

Nickel could potentially modulate osmoregulation (Bhatia et al., 2005). A high level of Ni in the plant tissue inhibits transpiration (Bazzaz et al., 1974). In spinach, Sn application increased the dry matter of both shoot and root (Müller et al., 2015) and increased growth in roots and plant height at lower concentrations of added Sn (0.2 mg/L or 1 mg/L) in pea and corn (Cohen, 1940).

Under drought, the application of P had beneficial effects on plant growth that included enhanced root growth (Singh and Sale, 1998), more leaf area and higher photosynthesis, and improved cell membrane stability and water relations (Kang et al., 2014). Similar findings on the morphological parameters (root length, leaf area, and dry weight of shoot and root) were observed on the drought-tolerant variety of chilli.

**Figure 8:**
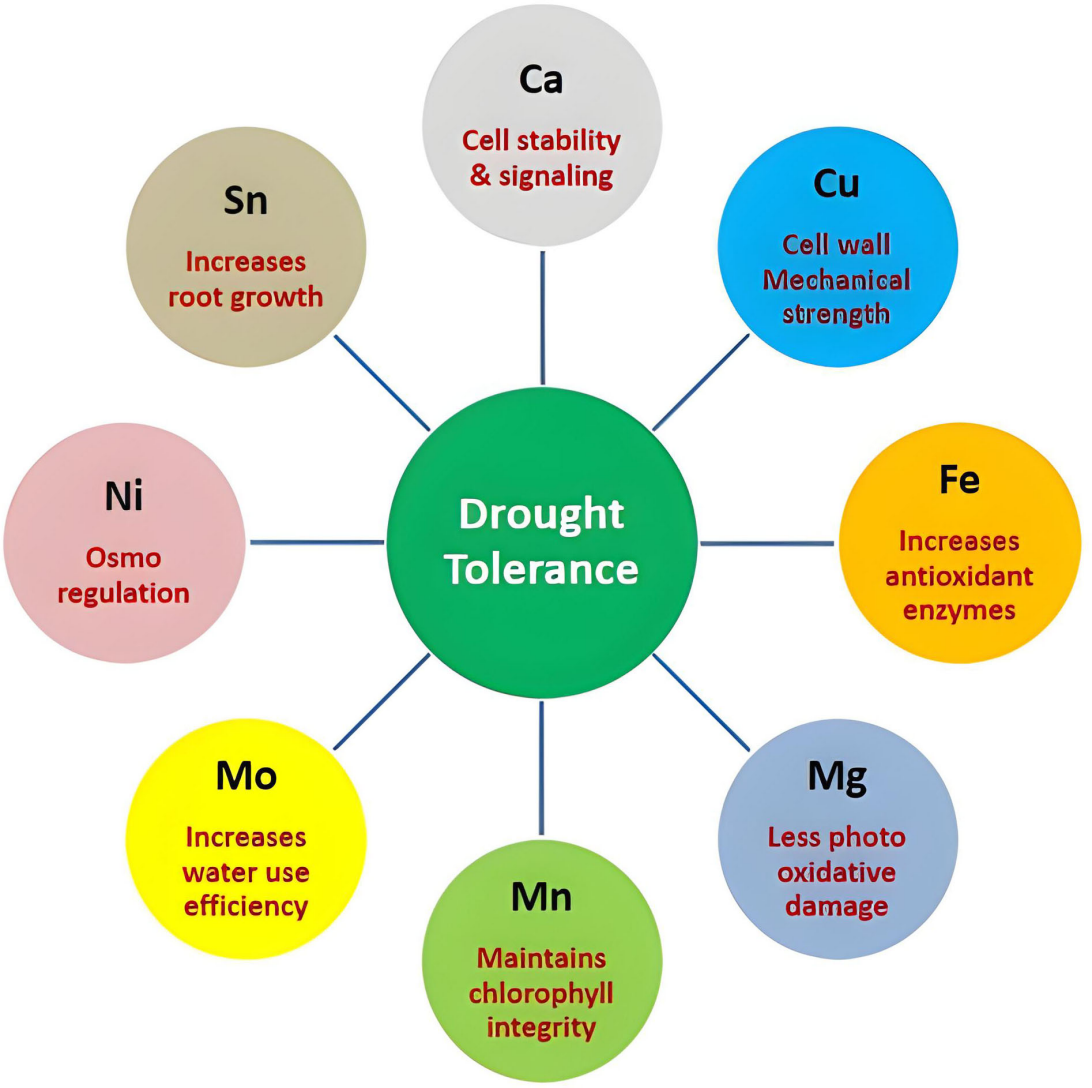
Functional roles of key ions mediating drought tolerance mechanisms in chilli cultivars.

Therefore, comparing ionome dynamics between tolerant and susceptible genotypes indicated Ca, Cu, Fe, Mg, Mn, Mo, Ni, and Sn as drought tolerance mediator ions (**Figure 8**).

## 5 Conclusion

The present research proved that ionomics is a useful and efficient approach for the characterization of drought-tolerant genotypes as well as the selection of adapted germplasm in chilli. The results showed that elements Ca, Cu, Fe, Mg, Mn, Mo, Ni, and Sn were critical in the regulation of drought tolerance and may be used as biochemical markers for stress resistance. These results provide an important foundation for breeding drought-tolerant varieties by simple, efficient methods that can be easily applied in the development of elite cultivars for this industrial and export-oriented crop. Under a climate change scenario, water scarcity is a major risk factor responsible for the decline in agricultural productivity, and the integration of modern breeding tools such as ionomics into pre-breeding pipelines provides an effective strategy for expediting the development of climate-resilient chilli varieties.

## 6 Future prospects

Future studies could include large and diverse germplasm panels with high-throughput ionome profiling and physiological phenotyping to discover new alleles for coordinated drought tolerance mechanisms. Combining this knowledge with genomic selection and marker-assisted breeding is likely to lead to elite chilli cultivars resistant to future climate variation. Morpho-physiological and molecular studies on individual drought tolerance mediator element-based crop responses will give a more in-depth understanding of the mitigation of drought stress.

## Data Availability

The original contributions presented in the study are included in the article/supplementary material. Further inquiries can be directed to the corresponding author.
